# Comparative genomic analysis of the odorant-binding protein family in 12 *Drosophila *genomes: purifying selection and birth-and-death evolution

**DOI:** 10.1186/gb-2007-8-11-r235

**Published:** 2007-11-08

**Authors:** Filipe G Vieira, Alejandro Sánchez-Gracia, Julio Rozas

**Affiliations:** 1 Departament de Genètica, Facultat de Biologia, Universitat de Barcelona, Av. Diagonal 645, Barcelona 08028, Spain; 2 Departamento de Neurobiología Molecular, Celular y del Desarrollo, Instituto Cajal, CSIC, Av. Dr. Arce 37, Madrid 28002, Spain

## Abstract

**Background:**

Chemoreception is a widespread mechanism that is involved in critical biologic processes, including individual and social behavior. The insect peripheral olfactory system comprises three major multigene families: the olfactory receptor (Or), the gustatory receptor (Gr), and the odorant-binding protein (OBP) families. Members of the latter family establish the first contact with the odorants, and thus constitute the first step in the chemosensory transduction pathway.

**Results:**

Comparative analysis of the OBP family in 12 *Drosophila *genomes allowed the identification of 595 genes that encode putative functional and nonfunctional members in extant species, with 43 gene gains and 28 gene losses (15 deletions and 13 pseudogenization events). The evolution of this family shows tandem gene duplication events, progressive divergence in DNA and amino acid sequence, and prevalence of pseudogenization events in external branches of the phylogenetic tree. We observed that the OBP arrangement in clusters is maintained across the *Drosophila *species and that purifying selection governs the evolution of the family; nevertheless, OBP genes differ in their functional constraints levels. Finally, we detect that the OBP repertoire evolves more rapidly in the specialist lineages of the *Drosophila melanogaster *group (*D. sechellia *and *D. erecta*) than in their closest generalists.

**Conclusion:**

Overall, the evolution of the OBP multigene family is consistent with the birth-and-death model. We also found that members of this family exhibit different functional constraints, which is indicative of some functional divergence, and that they might be involved in some of the specialization processes that occurred through the diversification of the *Drosophila *genus.

## Abstract 2

The comparative analysis of the odorant binding protein family in 12 Drosophila genomes allowed the identification of 595 putative family member genes and revealed insights into the evolution of this family in these species.

## Background

Olfaction plays a major role in the fitness of an organism, providing the odor and pheromone perception essential for its survival and reproduction. The olfactory system of insects has a great sensitivity and specificity. Indeed, the identification of odors is critical in a range of activities, including detection of food sources, egg-laying substrates, mates, and predators, and it also facilitates communication and social coordination. An understanding of the evolution of genes involved in olfactory perception may therefore provide insights into the role played by adaptation at the molecular level. In fact, positive selection has been proposed to explain the evolution of a number of olfactory system genes, both in insects and vertebrates (for example [1-7]).

In *Drosophila*, the olfactory system is restricted to the third antennal segment and the maxillary palp [8]. The chemical signals (the odorants) are detected in the aqueous lymph of the chemosensory hairs, the olfactory sensilla, where there exists a high concentration of odorant-binding proteins (OBPs). The OBPs are small (10 to 30 kDa), globular, and rather abundant water-soluble proteins that are synthesized and secreted by auxiliary cells surrounding the olfactory receptor neurons [9-12]. These molecules are characterized by a specific protein domain that comprises six *α*-helices joined by three disulfide bonds [13-15]. Although the full function of the OBPs is not well established, it is believed that they may act as molecular carriers that transport odorants and deliver them to the olfactory receptors (Ors), located on the sensory neurons [10,16-20]. In addition, the OBPs might play active roles in the olfactory code [21-26] as well as in stimulus inactivation [18,27,28]. Moreover, expression analyses in a number of insect species indicate that OBPs are not restricted to the olfactory tissues and in fact may also participate in other physiologic functions (for review [29]). Despite the fact that some OBPs from vertebrates and insects have equivalent functions, they are not homologous and actually differ in structure and size [30].

In insects, the OBP genes constitute a quite old multigene family, with their most recent common ancestor (MRCA) tracing back at least to the origin of insects. In spite the high conservation at the protein structure level [15], OBP members are fairly divergent. Based on amino acid similarity, the Lepidoptera OBP gene family was subdivided into three subfamilies: the general odorant-binding proteins, which probably bind and transport general odorants; the pheromone-binding proteins, which were proposed to be specialized in pheromone perception; and the antennal-binding protein X, with a function that is still to be elucidated. Although other insects have some OBP sequences that are closely related with those of Lepidoptera, essentially they cluster following the species phylogenetic relationships (for review [29]).

In *Drosophila melanogaster*, the OBP multigene family comprises 51 members [31], which is a number quite similar to that of the Or multigene family (62 Or genes [32]). Despite this, OBP and Or genes exhibit a markedly different genomic distribution; whereas OBP genes are mainly organized in clusters, Or members are scattered across the whole genome. The recent availability of the genome sequences of the malaria mosquito *Anopheles gambiae *and the honeybee *Apis mellifera *provided insight into the genomic organization of members of the three major multigene families that are involved in the peripheral events of chemosensory perception [7,33,34]: the OBPs, Ors, and gustatory receptors (Grs). Interestingly, the gene repertoire number across families is similar in *D. melanogaster *(51, 62 and 68 for OBPs, Ors and Grs, respectively) and in *Anopheles gambiae *(66, 79 and 76 for OBPs, Ors and Grs, respectively). In *Apis mellifera*, however, the three families expanded differently (21, 170 and 10 for OBPs, Ors and Grs, respectively). These three species nevertheless share a common distribution pattern: OBP members are clustered, whereas Ors and Grs are more scattered across the genome. The organization of the OBP repertoire also differs among species. In *Anopheles*, OBP clusters are smaller and more dispersed than in *D. melanogaster *and include members of all previously identified subfamilies but also members of a new atypical class [33]. In *Apis mellifera *the OBP genes are also organized in genomic clusters [7] despite the reduced number of genes (21 members); the honeybee genome nevertheless contains members of only two OBP subfamilies.

The recent availability of the whole genome sequence for 12 species of the *Drosophila *genus represents a first opportunity to conduct a fine-scale molecular evolution analysis in a complete multigene family on a suitable time scale. Here, we analyzed the complete OBP gene repertoire in these *Drosophila *spp. In particular, we reconstructed the evolutionary history of these genes to determine the global mode of evolution of the OBP multigene family, and to gain insight into the selective forces that drive the evolution of these olfactory-specific genes. We show that the OBP multigene family evolves in accordance with the birth-and-death model, and we estimate the number of gene gains and losses in each lineage and the birth-and-death rates. Additionally, we report significant insight into the origin and fate of the OBP duplicate copies as well as the distribution of functional constraints among generalist and specialist *Drosophila *spp.

## Results

### Odorant-binding protein multigene family

**Figure 1:**
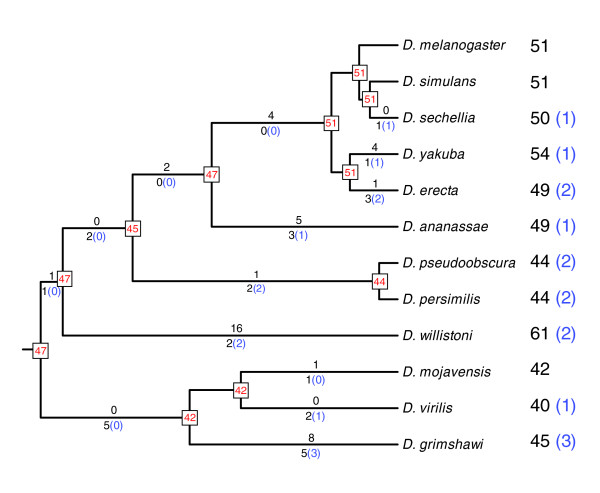
OBP gene repertoire in the 12 *Drosophila *spp. Values on the right part indicate the number of putative functional genes and pseudogenes (in parenthesis). Values in red indicate the inferred number of genes at the ancestral nodes of the phylogenetic tree. Values above the branches show the number of gene gains (gene duplications events), and below the branches X(Y) represent the total number of losses (deletions plus pseudogenization events [X]) and pseudogenization events (Y). OBP, odorant-binding protein.

Available comparative analysis freeze 1 annotations and current searches using TBLASTN and PSI-BLAST allowed the identification of a total of 595 OBP genes (Figure 1 and Additional data file 4) that encode putative functional and nonfunctional OBPs across the genome of the 12 *Drosophila *spp. This number includes orthologs of the 51 genes already annotated in *D. melanogaster *[31] as well as a number of gene gains in specific *Drosophila *lineages (Figure 1). Overall, we identified 54 OBP orthologous groups, including orthologs and co-orthologs, among the *Drosophila *spp. (see Additional data file 4); 34 of these groups include at least one member in all 12 *Drosophila *spp. Altogether, 580 OBP genes appear to be functional, having neither frameshift nor premature stop mutations; five of them nevertheless have an incomplete DNA sequence. In addition, 15 members probably represent pseudogenes because they are defective in length, lack splicing sites, or have incorrect transcription termination signals (premature stop codons). We also analyzed four additional OBP-like members, namely the chemosensory proteins (CSPs), which might represent a new class of OBP [35]. The CSP genes have orthologs in all 12 *Drosophila *genomes. Nevertheless, one *D. ananassae *member appears to be a pseudogene.

Overall, we inferred 43 gene gains and 28 gene losses (15 deletions and 13 pseudogenization events) across the evolution of the 12 species (Figure 1). Interestingly, deletions and pseudogenization events were not randomly distributed. Actually, 11 of such pseudogenization events were in external branches of the phylogeny, whereas the remaining two were in the internal branch leading to the short *D. pseudoobscura *and *D. persimilis *lineages (*χ*^2 ^test; *P *= 0.037 and *P *= 0.004 using all 13 pseudogenization events, and excluding events in *D. pseudoobscura *and *D. persimilis *lineages, respectively). We also found that the relation between extant genes and gene duplication events were not evenly distributed among the *Drosophila *chromosome arms (known as Muller elements; Fisher's exact test, *P *= 0.011). Indeed, 37 out of 43 inferred duplication events were located on Muller's C element, whereas this element harbors just about half of the OBP genes (29 genes in *D. melanogaster*). This feature therefore suggests that the gene duplication rate is higher in high-density OBP gene regions. As expected, new duplicate genes are much more likely to be lost because of a pseudogenization event than are older ones (*χ*^2 ^test; *P *= 9.00 × 10^-12^, for duplication events on external branches of the tree).

**Figure 2:**
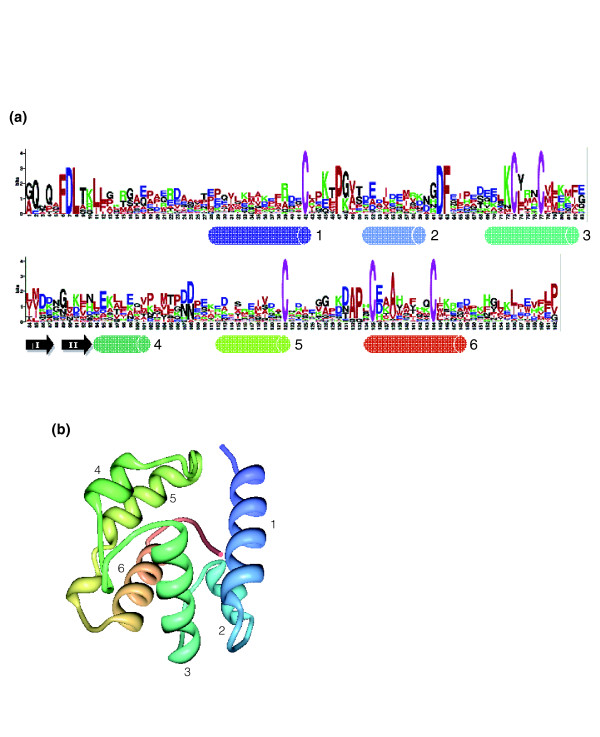
OBP sequence logo and three-dimensional structure. **(a) **Degree of amino acid sequence conservation [98] along the odorant-binding protein (OBP) sequences in phylogenetic subfamilies II and III. The locations of the *α*-helices were determined by homology with LUSH (*DmelObp76a *gene from OBP subfamily III). *α*-Helices are depicted as cylinders and beta sheets as bold arrows. **(b) **Three-dimensional structure of the LUSH Protein (PDB ID: 1OOI) [99], visualized using the Molecular Biology Toolkit platform [100]. The color ramp ranges from blue (amino-terminal) to red (carboxyl-terminal).

All predicted OBP proteins have the hallmarks feature of the family: the *α*-helix pattern, the highly conserved cysteine profile, the typical basic secondary-structure (Figure 2), and probably a globular water-soluble nature. Moreover, we also identified the signal peptide sequence region on the amino-terminus of the protein in nearly all OBPs. However, these genes are rather diverse (the overall amino acid divergence per site is 3.01, about 17% amino acid identity), differing in the numbers of amino acid residues, *α*-helices, and putative disulfide bonds. On this basis, the 58 *Drosophila *OBP-CSP orthologous groups fall into three of the four groups described by Zhou and coworkers [36]: 41 in the classic OBPs (six cysteines, four to six *α*-helices, and 111 to 280 amino acids, excluding dimer and minus-C OBP genes), 13 in the plus-C class (12 cysteines and one proline, six to eight *α*-helices, and 170 and 245 amino acids), and four in the CSP (four cysteines, six *α*-helices, and 112 to 155 amino acids) group. The atypical OBP group (nine to ten cysteines), described in *A. gambiae *[33], is not represented in the *Drosophila *genus. The average amino acid divergence per site within orthologous groups is 0.39 (71% amino acid identity), ranging from 0.15 to 1.02.

From the OBP members present only in extant species, we estimated the OBP birth-and-death (*λ*) rate per copy and million years [37,38] to be 0.0053 to 0.0081, using *Drosophila *divergence times from Tamura and coworkers [39] and Russo and colleagues [40], respectively. Information of the inferred numbers of gene gain and loss events in each branch of the phylogeny (Figure 1) allows us to estimate the birth-and-death rates separately (equations 1 and 2; see materials and methods, below). Using the same divergence times, the birth rate (*β*) is 0.0024 to 0.0040, whereas the death rate (*δ*) is 0.0016 to 0.0026.

### Chromosomal distribution of odorant-binding protein genes

**Figure 3:**
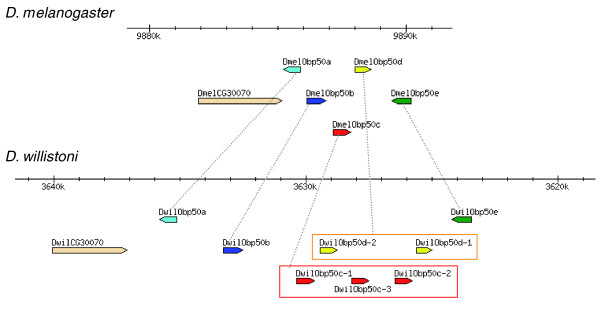
Chromosomal organization of OBP cluster 2 in *Drosophila melanogaster *and *D. willistoni*. Also see Additional data file 1. OBP, odorant-binding protein.

It has been shown that the distribution of OBP genes in insect genomes is not random and that they usually occur in genomic clusters [7,31,33]. This feature was also observed across the genomes of the 12 *Drosophila *spp., in which 69% of OBP genes are arranged in clusters. Operationally, we consider that *n *closely linked OBP genes form a genomic cluster if they are arranged within a genomic region of 10(*n *- 1) kilobases (the average gene density in *Drosophila *is approximately of one gene per 10 kilobases), or within a genomic region having fewer than *n *- 1 non-OBP genes. In the 12 genomes, we identified ten clusters of two to six OBP genes (Additional data files 1 and 5; also see Figure 3). Although the genome assembly is not fully completed for all *Drosophila *spp., we were able to determine that these clusters, including the direction of transcription of its members, are conserved across the 12 species. The only two exceptions are the *DmojObp99c *gene (a member of the cluster 10, which appears isolated in *D. mojavensis*) and the *DwilObp56e *member (transcription of which occurs in the opposite direction in *D. willistoni*). Although gene clusters have changed their relative physical position across species, they are always maintained in the same Muller element, with the sole exception corresponding to one case in *D. ananassae*. This feature points to chromosomal inversions as being the main mechanism responsible for these rearrangements. As previously observed in *D. melanogaster *[31], the locations of OBP genes across *Drosophila *chromosomes are not randomly distributed, with the majority of OBP genes (82%) being in Muller's C and E elements.

### Phylogenetic analysis

**Figure 4:**
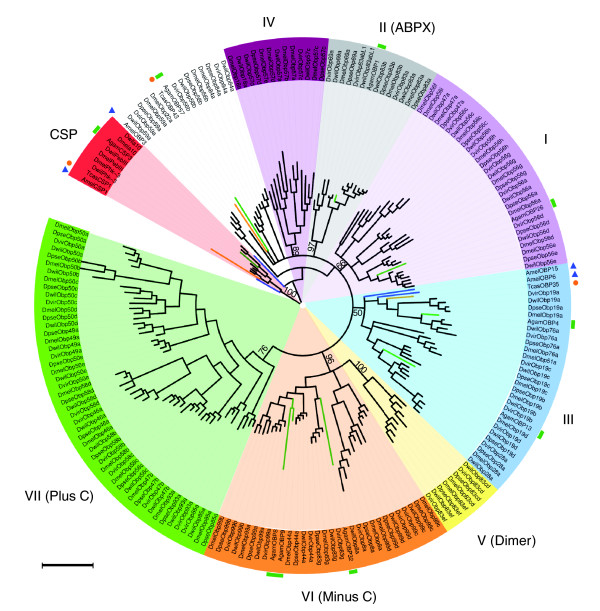
Phylogenetic relationships of the OBP proteins. The unrooted tree includes odorant-binding protein (OBP) sequences from *Drosophila *(for clarity the tree only includes sequences from *Drosophila melanogaster*, *D. pseudoobscura*, *D. virilis*, and *D. willistoni*), *Anopheles gambiae *(green rectangles), *Apis mellifera *(blue triangles), and *Tribolium castaneum *(orange circle). The OBP (I to VII) and chemosensory protein (CSP) phylogenetic subfamilies are shadowed by different colors. The scale bar represents 1 amino acid substitution per site. The tree was displayed using the iTOL web server [101]. ABPX, antennal-binding protein X.

We conducted a phylogenetic analysis including the OBP proteins from *Drosophila *spp. and representatives of the OBP subfamilies from other insects (*Anopheles gambiae*, *Apis mellifera*, and *Tribolium castaneum*). The results (Figure 4) clearly show that orthologous groups of *Drosophila *share a MRCA more recent (nearly all posterior probabilities are greater than 99%) than that of the paralogous copies. Hence, OBP genes have evolved independently since their origin by gene duplication. This result is in accordance with the negative outcome of the gene conversion analysis (results not shown). The phylogenetic analysis shows that all CSP members form a monophyletic clade, tracing the OBP-CSP genes back to the origin of the arthropods [29]. The analysis also confirms the previously proposed phylogenetic subfamilies (classic and plus-C). However, the classic OBP subfamily [33] can be further subdivided into other phylogenetic clades (Figure 4 and Additional data file 5), including those described by Hekmat-Scafe and coworkers [31] and Zhou and coworkers [41]. The two dimer OBP genes (*Obp83cd *and *Obp83ef*) are composed by two consecutive OBP domains, and probably originated from a gene duplication event or by fusion of two linked OBP genes; regardless, each component would belong to the minus-C group. In general, the exon/intron gene structure is relatively well conserved within phylogenetic groups, supporting the evolutionary meaning of the previous classification (Additional data file 2). The average amino acid divergence within each phylogenetic clade is 1.89 (about 33% amino acid identity). In addition, we found a significant association between phylogenetic subfamilies and genomic clusters (Fisher's exact test, *P *< 1 × 10^-15^); specifically, the OBP members of a given genomic cluster tend to be phylogenetically related.

### Natural selection on the odorant-binding protein multigene family

**Figure 5:**
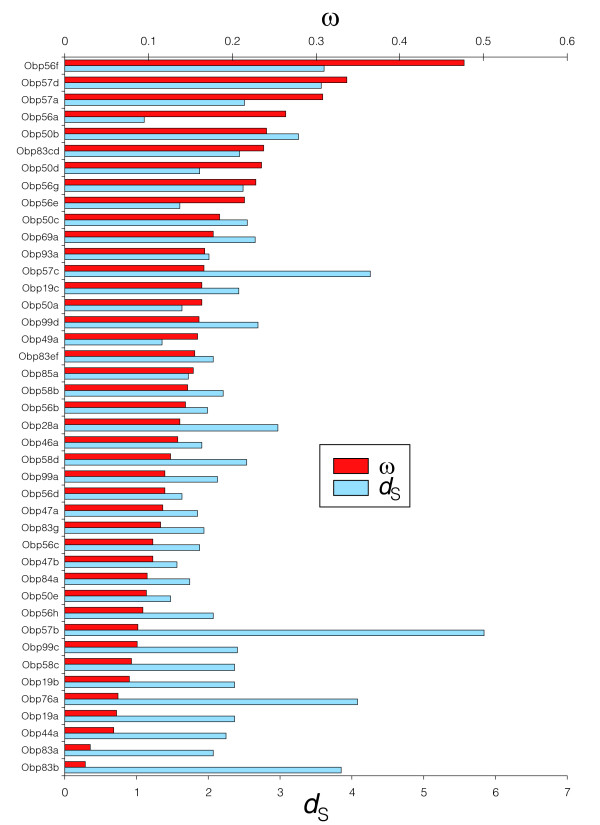
Global *d*_S _and *ω *values across the OBP genes under the M3 (*K *= 2) model. The analysis was conducted using information of only the six species of the *melanogaster *group of *Drosophila*. *d*_S_, synonymous substitution rate; OBP, odorant-binding protein; *ω*, nonsynonymous substitution rate/*d*_S_.

**Figure 6:**
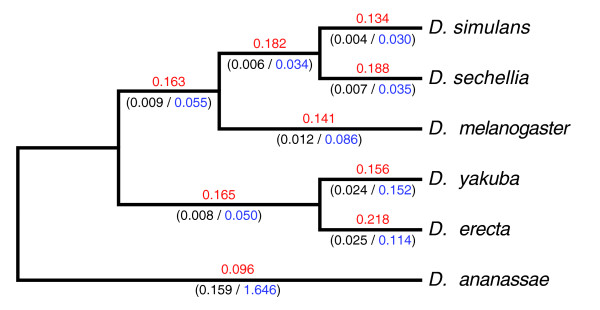
Selective constraints on the six species of the *melanogaster *group of *Drosophila*. Numbers above branches (in red) show the estimates of the *ω *parameter (*ω *= nonsynonymous substitution rate [*d*_N_]/synonymous substitution rate [*d*_S_]). Numbers below branches indicate the *d*_N _(in black) and *d*_S _(in blue) values, respectively. The *ω *values were calculated applying the free ratio (FR) model on the concatenated multiple alignment (set of 42 orthologous groups).

We studied the selective forces acting on the OBP gene family in the six species of the *Drosophila melanogaster *group (see Materials and methods, below); the analysis was conducted using the orthologous groups with a full coding DNA sequence in each of the six species (42 multiple sequence alignments; Additional data file 6). Overall, the maximum likelihood estimates of the *ω *parameter are relatively low (average *ω *= 0.153), suggesting that purifying selection is the predominant force acting on the evolution of the OBP gene family (Figure 5). Interestingly, we detected heterogeneity in the *ω *values across OBP genes (*P *= 2.1 × 10^-10^), suggesting some functional constraint differences. The likelihood ratio test contrasting the one ratio (M0) and the free ratio (FR) models (branch-model analysis) rejects the null hypothesis in 14 cases (four cases after controlling for the false discovery rate); therefore, these genes are probably evolving at different functional constraints across the *Drosophila *genus. The *D. sechellia *lineage exhibits greater *ω *values in three of these four genes, namely *DsecObp19b*, *DsecObp56c*, and *DsecObp58b *(see below). Interestingly, the FR model applied to the concatenated data (the multiple sequence alignments of the 42 orthologous groups) fits the data significantly better than does the M0 model (*P *= 4.04 × 10^-20^), revealing an episodic mode of evolution; that is, the overall selective constraint levels fluctuate across the *melanogaster *group lineages (Figure 6).

We have also studied putative OBP evolutionary rate differences between specialist (*D. sechellia *and *D. erecta*) and generalist (*D. melanogaster*, *D. simulans*, *D. yakuba*, and *D. ananassae*) species across the *melanogaster *group of *Drosophila *[42-45], by assessing the fit of the M0spec model to the OBP data (see Materials and methods, below). This model fits the data significantly better than the M0 model (null hypothesis) for 17 OBPs (11 cases after controlling for the false discovery rate). For the total concatenated dataset, the M0spec model also fits the data significantly better than M0 (*P *= 9.42 × 10^-24^; Figure 6), whereas the FR model failed to reject it (*P *= 0.699). In fact, the *ω *ratios in the OBP genes in specialist lineages are significantly higher than those in the generalists (Paired Wilcoxon rank sum test, *P *= 0.0009), further supporting the hypothesis of a change in the selective constraints in these lineages [45].

The maximum likelihood analysis using codon evolution models allowing for heterogeneity in the distribution of the *ω *ratio along the amino acid sequence reveals significant results in most *Drosophila *OBP genes. As a matter of fact, there are two distinct classes of amino acid evolving positions (40 significant comparisons after controlling for the false discovery rate; M3 [*K *= 2] model, average *ω*_0 _= 0.033 and *ω*_1 _= 0.513). However, these tests failed to detect significant evidence of positive selection in OBP evolution across the *melanogaster *group (that is, the M1 and M7 models could not be rejected). This analysis applied to the concatenated data confirms that M3 model fits the data better than does M0 (*P *< 0.0001; 75% positions with *ω*_0 _= 0.038, and the rest with *ω*_1 _= 0.591).

**Figure 7:**
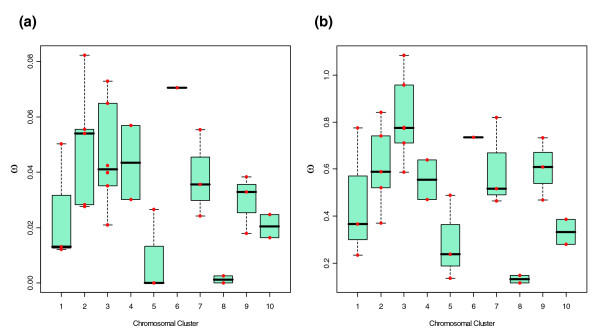
Distribution of the *ω *values (M3 (*K *= 2) model) across the OBP chromosomal clusters. The analysis was conducted using information of only the six *melanogaster *group species of *Drosophila *(42 orthologous groups). **(a) **Distribution of the estimated *ω*_0 _values. **(b) **Distribution of the estimated *ω*_I _values. OBP, odorant-binding protein.

Overall, we did not detect significant differences in the global *ω *values (M3 [*K *= 2] model) across Muller elements (sum of squares between groups [SSB] test, *P *= 0.706), genomic clusters (SSB test, *P *= 0.083), or phylogenetic subfamilies (SSB test, *P *= 0.118; Additional data file 3). Interestingly, the estimated *ω*_0 _and *ω*_1 _values differ across chromosomal clusters (SSB test, *P *= 0.021 and *P *= 0.057, respectively; Figure 7). Although close to the critical value, we did not find significant association between temporal gene expression pattern and phylogenetic groups or genomic clusters (Fisher's exact test, *P *= 0.070 and *P *= 0.146, respectively). However, this result should be interpreted with caution because the data on OBP expression patterns are still incomplete.

## Discussion

The availability of the complete genome sequence of 12 phylogenetically close *Drosophila *spp. represents a milestone in comparative genomics. The phylogenetically guided analysis of genome information can provide a fine description of the gene structure of the complete set of genes, and can clearly improve our knowledge of the evolution of *Drosophila *spp. Indeed, analysis of currently available genome information can provide insight into the major forces that shape gene family evolution. Here, we conducted an exhaustive gene-by-gene bioinformatic analysis, precisely identifying orthologous members and the number of gene gains and losses, which allows one to draw accurate evolutionary inferences. We show that, in the *Drosophila *genus the number of OBP genes is moderately variable (from 40 to 61 genes) across species diverging only about 40 to 60 million years [39,40], and that the MRCA of the 12 *Drosophila *species had 47 genes. Moreover, the species visibly differ with respect to pseudogenes; although the number of nonfunctional genes is nearly the same (from 0 to 3 copies per species), they represent different pseudogenization events, except for the two pseudogenes identified in the closest species *D. pseudoobscura *and *D. persimilis*. The 12 *Drosophila *spp. Nevertheless maintain the basic chromosomal organization of the OBP genes in clusters; however, although the relative physical position of clusters differs among species, they are always maintained within the same Muller element. This feature therefore points to chromosomal inversions as being the main mechanism responsible for chromosomal rearrangements [46-48].

Several models have been proposed to explain the global evolution of multigene families (for review [49]). There are three basic models. The divergent evolution model [50] postulates that duplicate copies diverge in a gradual manner and that new functions are acquired progressively. The concerted evolution scenario [51] proposes that gene family members evolve in a concerted manner through gene conversion, unequal crossover, or gene amplification [52]. More recently proposed [53] is the birth-and-death model of gene family evolution, which states that the new genes are created by gene duplication and are lost by deletions or become nonfunctional accumulating deleterious mutations. Under the latter model, different gene duplicates would differ in the times that they are maintained in the genome. The controversy over the relative importance and interplay of these multigene family evolution models currently remains active [49,54]; two critical limiting issues are the lack of DNA sequence data from the complete set of genes and pseudogenes in multiple genomes, and the partial knowledge of the gene conversion mechanism and therefore of its significance.

Analysis of the whole set of OBP genes and pseudogenes in the complete genome of the 12 *Drosophila *spp. clearly points to birth-and-death as being the major model for the evolution of the OBP multigene family. First, the phylogenetic analysis shows that orthologous groups share a MRCA more recent than that of paralogous groups (the average amino acid divergence within orthologous groups is much lower than estimates, including orthologous and paralogous), in addition to the lack of evidence supporting gene conversion. Second, orthologous copies fit very well the accepted phylogeny of the species. Third, we detected a number of gene gain and loss events in numerous lineages of the phylogeny. Fourth, we also identified several nonfunctional members (pseudogenes). Therefore, OBP genes would evolve independently from their origin by gene duplication until their loss by deletion or transiently as a pseudogene.

Under the birth-and-death model, the new duplicate genes are eventually lost from the genome by two basic processes: by a deletion or via pseudogenization. Our study shows that all pseudogenization events, except for the two inferred in the ancestral branch leading to the short *D. pseudoobscura *and *D. persimilis *lineages, occurred in terminal branches. It is most likely that the failure to detect pseudogenes on internal phylogeny branches is caused by the short half-life of pseudogenes (the elapsing time before a pseudogene can no longer be recognized as a member of its original sequence family is very short). Therefore, we cannot quantify the relative magnitude of the two processes. Nonetheless, the uneven distribution of deletions and pseudogenizations on internal and external branches of the phylogeny suggests that several gene losses detected as deletions were initially triggered by a pseudogenization event.

Our comparative genome analysis has also provided insights into the rates of the origin and loss of duplicate genes. In particular, we estimated the birth-and-death rate by two approaches: the stochastic birth-and-death process, which uses information of the number of genes in extant species and assumes equal rates of gene gain and loss [37,38]; and comparative genome analysis of the inferred number of gene gain and loss on each phylogeny branch and those inferred at the internal nodes (see Materials and methods, below). We estimated the birth-and-death rates as *β *= 0.002 to 0.004 and *δ *= 0.002 to 0.003 per gene and million years. These estimates are slightly lower than those obtained using the method proposed by Hahn and coworkers [37] (*λ *= 0.005 to 0.008);, the latter method, however, assumes equal gain and loss rates. Present OBP estimates are higher than the average value for the whole *Drosophila *genome (*λ *= 0.0013 [55]) although similar (or lower) to the estimates obtained for the two other major olfactory multigene families of *Drosophila*, namely the Ors (*λ *= 0.006 to 0.009) and the Grs (*λ *= 0.011 to 0.015). (These estimates were derived using the numbers of genes identified by McBride and Arguello [56] and those identified by Gardner and Ritchie [personal communication].) OBP birth rates are quite similar to previous estimates for the complete set of gene families in *Drosophila *(*β *= 0.001 to 0.002 [57,58]). However, these estimates are not completely comparable because the methodological approach used by Lynch and Conery [57,58] is quite different from our approach in that they made use of single-genome information. We also show that high-density tandem OBP gene regions are more likely to generate new duplicates. Therefore, a given gene family might present different birth (or birth-and-death) rates across the genome. As more genome information becomes available, it will be possible to determine whether the birth-and-death rates differ in gene families with different function, chromosomal locations, dissimilar gene sizes, or in different group of species, and whether they correlate with gene-specific functional importance [59]. These studies will undoubtedly provide valuable insight into the molecular evolution and biologic importance of multigene families.

The present study also provides significant clues as to the origin and fate of duplicate genes. We show that the majority of gene gains occur in extant chromosomal clusters, suggesting that gene duplications are mostly produced in tandem by unequal crossing over. Furthermore, the highly significant relationship between chromosomal clusters and phylogenetic groups would indicate that OBP members evolve gradually from their origin in existing clusters. It is known that transposable element-rich regions could generate these 'gene factories' by increasing the levels of unequal crossing over [60-62]. Although we did detect a number of repetitive elements neighboring most of the genome clusters, no confident conclusion could be drawn. Further analysis of the relative distribution of these repetitive elements will provide more information about the origin of OBP gene duplicates and genome clusters.

We found that OBP genes exhibit high functional constraints, with an average *ω *value of 0.153, and confirmed that the results obtained in two individual members are a general feature of the family [63,64]. In spite of the fact that the selective constraint levels are not clearly associated with phylogenetic groups, they differ both among individual genes and across chromosome clusters. This feature supports the contention that, concurrent with the sequence divergence, duplicates copies would also diverge functionally. Although OBP members would essentially maintain the same global function, they would probably acquire subtle functional differences (a micro-functionalization [62]), perhaps in their gene expression levels or in their ligand-binding specificity or affinity properties. In addition, Andronopoulou and coworkers [65] have demonstrated that some *Anopheles *OBPs might form homodimers and specific heterodimers, suggesting a high combinatorial complexity that will allow for new binding or kinetic properties (also see Sánchez-Gracia and coworkers [63]). Hence, small differences in the number and pattern of protein-protein OBP interactions might have an appreciable functional meaning and might underlie the observed functional constraint differences [66,67]. In this context, it is suggestive that dimmer OBPs, which might have been originated from a gene duplication event followed by in-frame fusion, produce a single-chain multidomain protein that retains structural features of the original dimeric unit.

The present estimates of the protein evolutionary rates at the OBP genes are in contrast to the strong conservation pattern of genome clusters across the genus. However, this feature does not occur in the Or and Gr gene families, which have a comparable number of genes [56,68,69] (Gardner and Ritchie, personal communication), suggesting the action of some mechanism that actively prevents their break. Indeed, genes belonging to the same cluster might exhibit a spatiotemporally coordinated expression. For instance, in *D. melanogaster *some OBP genes are co-expressed either in the same developmental stage or in the same local region of the chemosensory organs [31,70]. Although we did not detect clear evidence that genes on the same cluster are expressed at particular developmental stages, the incomplete gene expression data precludes us from drawing any firm conclusion. To shed light on this issue, it is essential to determine how the expression patterns correlates with genomic gene organization in these olfactory system gene families.

The evolutionary analysis of the complete set of genes in a family involved in the response to environmental chemicals is also very attractive because they may be able to provide insights into the selective pressures that result from changes in the species 'lifestyle' during and after speciation. Here we find that OBP genes in specialist lineages (those that recently underwent a host speciation episode) evolve at significantly higher *ω *rates than do generalists. Consequently, either purifying selection is more relaxed in several OBP genes, probably caused by loss (or partial loss) of function during the specialization process, or positive selection acted throughout this process. McBride and Arguello [56] also found a significant increase in the evolutionary rate at the Ors and Grs in the *D. sechellia *and *D. erecta *lineages; this study detected a genome-wide increase in the amino acid fixation rate in this species, although it was lower than that observed at the receptor repertory. Because the genome-wide higher *ω *values detected in the specialist species could reflect some demographic changes, we have also conducted in the OBP family the same analysis as McBride and Arguello [56] did, using the same genome-wide set of genes. We also found that the OBP repertory in specialist species has evolved under lower functional constraints (higher *ω *values) than the genome-wide trend (the median difference between specialists and generalists *ω *for the OBP family [0.0556] is significantly greater than that for genes across the genome [0.0087]; *P *= 0.0031). This feature, jointly with the birth-and-death evolution pattern similarity, suggests that these two olfactory system multigene families might have co-evolved in response to ecologic changes across the *Drosophila *genus. Therefore, it would be very interesting to establish the precise contribution of the OBP gene family to this specialization process, and to identify the specific members involved in this phenomenon. This knowledge will provide fundamental insight into the roles played by the various selective forces in shaping patterns nucleotide variation associated with host-switching or ecologic specialization processes.

## Conclusion

The *Drosophila *OBP multigene family has evolved under purifying selection and accommodates remarkably well to the birth-and-death model, with tandem gene duplication being the major mechanism for generation of new members. However, OBP genes exhibit different functional constraints, which are indicative of some functional diversification. The organization of OBP genes in genomic clusters is conserved across the 12 species; therefore, the formation of the OBP cluster architecture should have been originated before the split between *Drosophila *and *Sophophora *subgenera. This feature suggests the existence of co-regulated gene clusters for most OBPs in these genomes. Finally, we have showed that this olfactory system protein family has evolved more rapidly in specialist species of the *melanogaster *group of *Drosophila*, and thus it might be involved in processes of ecologic diversification.

## Materials and methods

### Identification of the odorant-binding protein genes in *Drosophila*

The sequence of the OBP genes of *Drosophila melanogaster *(release 4.3 [71]), *D. pseudoobscura *(release 2.0 [72]), *Anopheles gambiae *(release 2.29 [73]), and *Apis mellifera *(release 4.0 [74]) were downloaded from the National Center for Biotechnology Information [75] and Flybase [76]. The protein sequences of the OBP genes of *Tribolium castaneum *were obtained from Foret and Maleszka [7]. Genomic information, including the orthologous relationships, of the ten new *Drosophila *spp. plus the two previously sequenced (comparative analysis freeze 1 stage) was downloaded from the Assembly, Alignment and Annotation Wiki [77-79].

To identify putative non-annotated OBP members, we first conducted a TBLASTN search against the genome sequence of the 12 *Drosophila *spp. (threshold E-value of 10^-5^). For the analysis we used as query the amino acid sequence of all known OBP members from all species (including *A. gambiae *and *A. mellifera*). Putative missing genes were confirmed by the colinearity analysis of single-copy conserved genes. First, we conducted a dot-plot analysis using the zPicture program [80] between the genomic region surrounding the putative missing gene (about 10 to 80 kilobases in length) and the orthologous syntenic region of the phylogenetically closest *Drosophila *spp. Next, we searched by MegaBLAST (threshold E-value of 10) the trace archives (the raw genome sequence data) using as the query the nucleotide information surrounding the putative absent OBP member. This exhaustive gene-by-gene analysis allowed us to determine accurately the number of gene gains and losses in each branch of the phylogeny. To determine whether some already annotated gene model was in fact a non-annotated (or wrongly annotated) OBP member, we conducted a PSI-BLAST search (threshold E-value of 10^-3^) against the annotated proteins of *D. melanogaster *and *D. pseudoobscura *using as the initial query OBP representatives of the different subfamilies. However, this analysis failed to detect any new OBP. Moreover, we also studied the gene structure (the presence of start and stop codons and the signal peptide region) and the substitution pattern (nonsense or frameshift mutations), in order to verify whether the identified OBP gene was a pseudogene or an incorrectly annotated sequence. The new OBP annotations will be available from FlyBase.

### Odorant-binding protein gene nomenclature

The current nomenclature for the OBP gene names of *D. melanogaster *[31] is based on their cytological map position. For clarity and to gain insight into the orthologous relationships among OBP members, we propose for this gene family the nomenclature scheme used for the Or and Gr multigene families [56,68] (Gardner and Ritchie, personal communication). The first four letters indicate the *Drosophila *species (for instance, *Dsim *would indicate the *D. simulans *species) and the next letters the OBP gene name in *D. melanogaster *(for example, *DyakObp83a *is orthologous to *DmelObp83a*, which was named *obp83a *by Hekmat-Scafe and coworkers [31]). For unparalogous copies (gene duplications in non-*D. melanogaster *lineages) we add a hyphen and a number; for example, *DyakObp93a-1 *and *DyakObp93a-2 *are co-orthologs of *DmelObp93a *(both are orthologs of *DmelObp93a*). We use the letter 'L' (meaning 'like') to name genes that are closely related to *D. melanogaster *even though they are absent in this species (new genes); for example, *DvirObp57cL1 *indicates a new gene identified in *D. virilis *(absent in *D. melanogaster*) that arose from a duplication of an ancestor of *Obp57c*. Putative pseudogenes are named following the same schema adding the suffix 'P' (for 'pseudogene'); for instance, *DanaObp59aP *indicates that in *D. ananassae Obp59a *gene is a pseudogene.

### Birth-and-death rates

We estimated the global birth-and-death rate of the OBP gene family by applying two methods and using the divergence times from Tamura and Subramanian [39] and Russo and coworkers [40]. The method proposed by Hahn and colleagues [37] (implemented in the software CAFÉ [38]), which assumes an equal probability of birth (duplication) and death (deletion or pseudogenization), uses information regarding the number of genes in extant species. The birth and death rate (*λ*; per gene per million years) is estimated by maximum likelihood. We also estimated separately the birth (*β*) and death (*δ*) rates (per gene per million years) using both current estimates of the number of gene gains and losses in each branch of the phylogeny and the number of gene copies at the internal nodes. These rates were estimated as follows:

$$\beta =\sum_{i=1}^{n}\left(G_{i}/C_{i}\right)/t$$

$$\delta =\sum_{i=1}^{n}\left(L_{i}/C_{i}\right)/t$$

Where *n *is the total number of phylogenetic tree branches; *G*_*i *_and *L*_*i *_are the numbers of gene gains and losses, respectively, on each branch *I*; *C*_*i *_is the number of gene copies at the internal node of branch *I*; and *t *is the total time of the phylogeny (the sum of the periods for all phylogenetic branches).

### Multiple sequence alignments

We constructed multiple sequence alignments of both amino acid and nucleotide coding regions. Given the high substitution rate found at the signal peptide portion of the OBPs, the multiple sequence alignments of these regions might be, in most cases, unreliable. We therefore used the PrediSi program [81] to identify the signal peptide sequences; all of these regions were removed before conducting the final alignment. Paralogous OBP proteins were aligned by using the SPEM program [82], which uses predicted secondary structure information to conduct the alignment. To generate the multiple sequence alignments of the orthologous coding regions we first aligned the amino acid sequences using PROBCONS [83], and then we used this alignment to guide the nucleotide coding region alignment. We generated a multiple sequence alignments for each OBP gene member; for those genes with multiple annotated isoforms we used information of only the isoform shared across all 12 *Drosophila *spp.

### Evolutionary analysis

The MEGA 3.1 software [84] was used to estimate amino acid divergences under the JTT empirical amino acid substitution model [85] and applying the pair-wise deletion option. The MrBayes version 3.1.2 software [86] was used to infer the Bayesian phylogenetic tree under the Whelan-Goldman model of amino acid evolution [87] (this model was visited >99% of times by Markov chain Monte Carlo). The model was run with four chains for 5.5 million generations sampling from the posterior density every 1,000 generations. The temperature parameter was finally set at 0.025, because it led to a more efficient chain heating scheme. We discarded 20% of the sampled steps as burn-in. This phylogenetic tree was similar than that obtained under the neighbor-joining algorithm [88] (results not shown). We applied the approach described by Sawyer [89] to detect putative gene conversion events among paralogous amino acid sequences of a given species. The analysis was conducted using all OBP members and also separately for each phylogenetic subfamily.

We used the ratio *ω *= *d*_N_/*d*_S _(where *d*_N _and *d*_S _are the nonsynonymous and synonymous substitution rates, respectively) to analyze the selective pressures acting on OBP genes. Under strict neutrality, synonymous and nonsynonymous mutations will be fixed at identical rates, with the expected ratio (*ω*) being equal to 1. Purified selection against deleterious nonsynonymous mutations will cause the fixation of synonymous mutations at a faster rate (assuming that synonymous mutations are strictly neutral) than nonsynonymous mutations, and therefore *ω *will be less than 1. In contrast, only advantageous nonsynonymous mutations could be fixed in the population at a faster rate than synonymous mutations, and thus *ω *might be greater than 1. We restricted this analysis to the six *Drosophila melanogaster *group species because the synonymous positions in more divergent comparisons might be saturated, producing unreliable estimates of the *ω *parameter. This analysis was conducted using the *codeml *program from the PAML software package version 3.15 [90]. Because the phylogenetic relationship of *D. erecta *and *D. yakuba *with respect to *D. melanogaster *is controversial [91], we applied the three most supported tree topologies for these species; topology 1 assumes that *D. yakuba *and *D. erecta *are sister species, and topologies 2 and 3 regard *D. yakuba *and *D. erecta*, respectively, to be sister taxa relative to *D. melanogaster*. We report the results of only the best supported tree topology; in the few cases with discrepancies between models, we used the topology of the best supported tree under model M0 (one *ω *ratio for all lineages and all sites). Nevertheless, and so that our findings could be compared with those reported by McBride and Arguello [56], in the analysis comparing generalists and specialist species we reported the results based on topology 1.

We applied 'branch models' M0spec (with three different *ω *classes: one for specialist lineages, one for generalist lineages, and an additional *ω *class for the *D. ananassae*) and FR (free ratios; the *ω *parameter is estimated independently in each branch). We also applied 'site models', which allow for heterogeneous selective pressures across amino acid sites [92-94]: the neutral model (M1 model; assumes two site classes, with *ω*_0 _< 1 and *ω*_1 _= 1); the positive selection model (M2; which adds to the M1 model an additional site class with *ω*_2 _> 1); the discrete model (M3; which uses an unconstrained discrete distribution of *K *classes to model heterogeneous *ω *ratios across sites; here we used this model with *K *= 2 and *K *= 3); and the *β *distribution based models, M7 (which assumes a *β *distribution for *ω *among sites) and M8 (which adds to the M7 model one extra class of sites, with *ω*_1 _> 1). In addition, we applied the method described by Yang and Swanson [95] to assess whether global *ω *values (averaged across sites) differ among OBP genes. We run all PAML models with three different initial *ω *values to avoid local optima. To test a number of hypotheses dealing with the selective pressures governing the evolution of these genes, we contrasted pairs of nested models using a likelihood ratio test [96]. These analyses were conducted for each gene separately and by concatenating single OBP genes from certain groups (for example, all genes, genomic clusters, and phylogenetic clades). To control the false discovery rate for multiple tests, we applied the procedure of Benjamini and Hochberg [97] at the level of *q *= 0.05. We tested the distribution of *ω *values among groups (phylogenetic subfamilies or genomic clusters) using the SSB term of the ANOVA as statistic; the *P *value was obtained by the Monte-Carlo permutation test. For the association analysis of the *ω *values and gene expression, and because OBP gene expression data are incomplete, we conducted the analysis on only two groups [31,70]: those genes expressed exclusively in adult and those expressed in both larvae and adult.

## Abbreviations

CSP = chemosensory protein; MRCA = most recent common ancestor; OBP = odorant-binding protein; Or = olfactory receptor; SSB = sum of squares between groups.

## Authors' contributions

JR conceived of and supervised all research. FGV developed the bioinformatics tools. FGV and AS-G analyzed the data. JR and AS-G wrote the first version of the paper.

## Additional data files

The following additional data are available with the online version of this paper. Additional data file 1 illustrates the chromosomal location of the OBP genes and clusters in *D. melanogaster*. Additional data file 2 is a figure showing the intron-exon gene structure for the *Drosophila *OBP phylogenetic subfamilies. Additional data file 3 is a figure showing the distribution of the *ω *values estimated under the M3 (*K *= 2) model. Additional data file 4 is a table of all OBP and CSP genes, including some descriptive information and listing all orthologous groups. Additional data file 5 is a table listing the OBP phylogenetic subfamilies and chromosomal clusters. Additional data file 6 is a table including information on the OBP orthologous groups used in the PAML analysis.

## Supplementary Material

Additional data file 1 This figure illustrates the chromosomal location of the OBP genes and clusters in *D. melanogaster*. Click here for file

Additional data file 2 This figure shows the intron-exon gene structure for the *Drosophila *OBP phylogenetic subfamilies. Exons are depicted in black; intron phases 0, 1 and 2 are represented in green, yellow and red, respectively. The scale bar represents 1 amino acid substitution per site. Click here for file

Additional data file 3 This figure shows the distribution of the *ω *values estimated under the M3 (*K *= 2) model. The analysis was conducted using information of only the six *melanogaster *group species of *Drosophila *(42 orthologous groups). (A) Estimated *ω *values across phylogenetic groups. (B) Estimated *ω *values across chromosomal clusters. Click here for file

Additional data file 4 Presented is a table of all OBP and CSP genes, including some descriptive information and listing all orthologous groups. Click here for file

Additional data file 5 Presented is a table listing the OBP phylogenetic subfamilies and chromosomal clusters. Click here for file

Additional data file 6 Presented is a table with information of the OBP orthologous groups used in the PAML analysis. Click here for file
