# Supplementary material for: Comparative genomic analysis of the odorant-binding protein family in 12 Drosophila genomes: purifying selection and birth-and-death evolution
Source: Genome Biol. 2007 Nov 8;8(11):R235. doi: 10.1186/gb-2007-8-11-r235 (PMC2258175; doi:10.1186/gb-2007-8-11-r235)
Supplement: Additional data file 1 — This figure illustrates the chromosomal location of the OBP genes and clusters in D. melanogaster. [file gb-2007-8-11-r235-S1.ppt]

## Slide 1
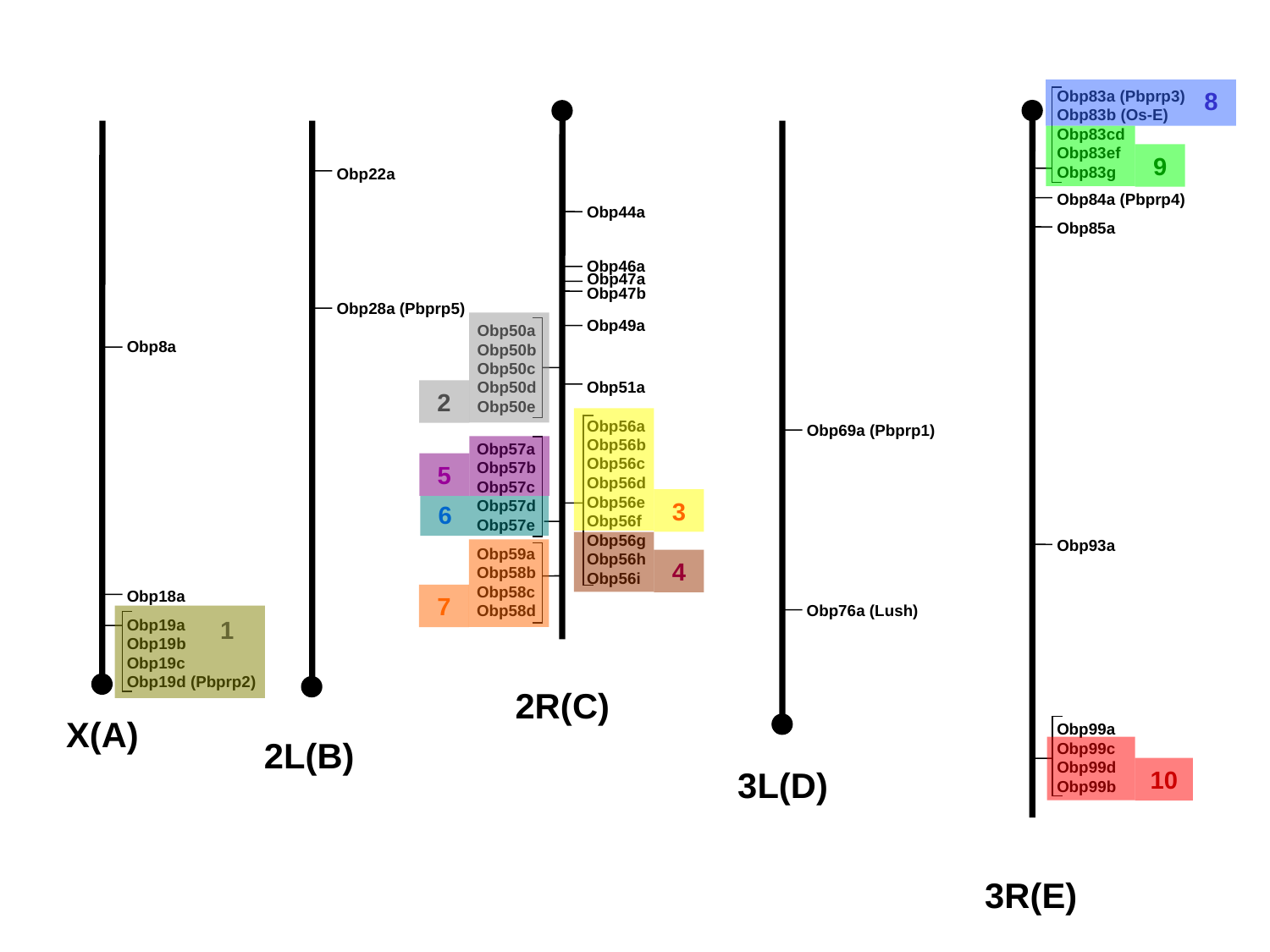

Obp83a (Pbprp3)
Obp83b (Os-E)
Obp83cd
Obp83ef
Obp83g
3R(E)
Obp84a (Pbprp4)
Obp85a
Obp93a
Obp99a
Obp99c
Obp99d
Obp99b
2R(C)
Obp44a
Obp46a
Obp47a
Obp47b
Obp49a
Obp50a
Obp50b
Obp50c
Obp50d
Obp50e
Obp51a
Obp56a
Obp56b
Obp56c
Obp56d
Obp56e
Obp56f
Obp56g
Obp56h
Obp56i
Obp57a
Obp57b
Obp57c
Obp57d
Obp57e
Obp59a
Obp58b
Obp58c
Obp58d
X(A)
Obp8a
Obp18a
Obp19a
Obp19b
Obp19c
Obp19d (Pbprp2)
2L(B)
Obp22a
Obp28a (Pbprp5)
3L(D)
Obp69a (Pbprp1)
Obp76a (Lush)
8
9
2
5
3
6
4
7
1
10
