# Supplementary figures and images for: Comparative genomic analysis of the odorant-binding protein family in 12 Drosophila genomes: purifying selection and birth-and-death evolution
Source: Genome Biol. 2007 Nov 8;8(11):R235. doi: 10.1186/gb-2007-8-11-r235 (PMC2258175; doi:10.1186/gb-2007-8-11-r235)

## Slide 1
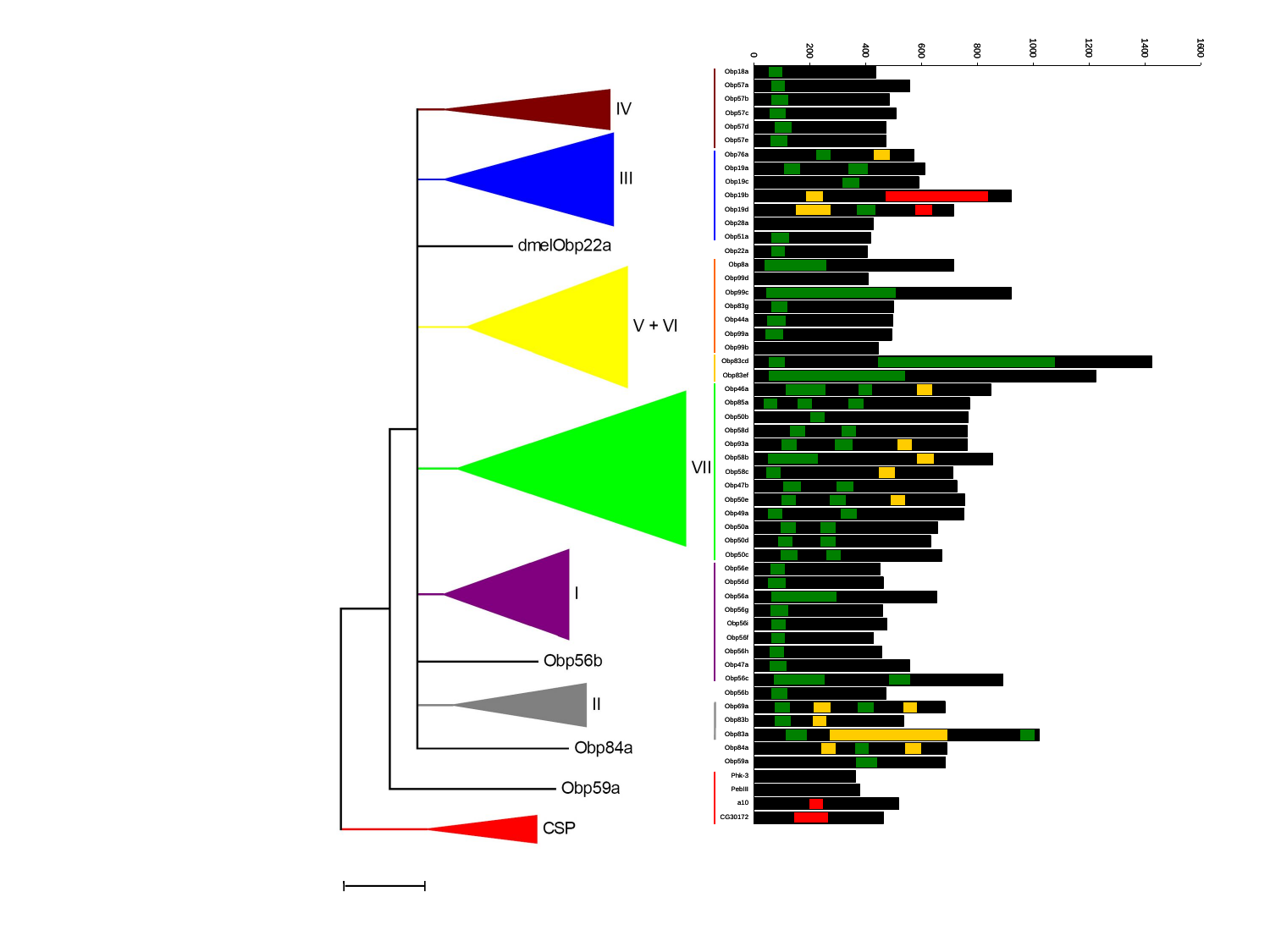

Supplement: Additional data file 2 — This figure shows the intron-exon gene structure for the Drosophila OBP phylogenetic subfamilies. Exons are depicted in black; intron phases 0, 1 and 2 are represented in green, yellow and red, respectively. The scale bar represents 1 amino acid substitution per site. [file gb-2007-8-11-r235-S2.ppt]
